# Hydrocéphalie sur thrombophlébite du sinus sagittal supérieur par ostéite de la voûte à *Aspergillus fumigatus* sur terrain immunocompétent

**DOI:** 10.11604/pamj.2018.31.97.14251

**Published:** 2018-10-09

**Authors:** Romuald Kouitcheu, Dominique N'Dri Oka, Guy Varlet

**Affiliations:** 1 Service de Neurochirurgie CHU Yopougon, Abidjan, Côte d’Ivoire

**Keywords:** Aspergillus fumigatus, hydrocéphalie, ostéite, thrombophlébite, Aspergillus fumigatus, hydrocephalus, osteitis, thrombophlebitis

## Abstract

Les auteurs rapportent dans une observation, une association pathogénique inhabituelle de lésions crânio-encéphaliques. Elle se caractérise par l'association d'une ostéite de la voûte à *Aspergillus fumigatus*, d'une thrombophlébite sous-jacente, elle-même compliquée d'une hypertension intracrânienne par hydrocéphalie. Nous rapportons le cas d'un homme de 43 ans, sérologie VIH (virus d'immunodéficience humaine) négative avec une notion d'infection multi-récidivante du scalp frontal. Ce patient a été traité avec succès par une dérivation du liquide cérébrospinal (LCS), du Kétoconazole et de l'héparine de bas poids moléculaire. Nous discutons à la lumière de la littérature les différents aspects physiopathologiques et de la prise en charge de cette exceptionnelle association pathogénique.

## Abstract (en)

This case study presents an unusual pathogenic association among several cranioencephalic lesions characterized by the association of osteitis of the cranial vault, due to Aspergillus fumigatus, with underlying thrombophlebitis complicated by intracranial hypertension resulting from hydrocephalus. The study involved a 43-year old HIV (human immunodeficiency virus) negative man with multi-recurrent infection of the frontal scalp. The patient was successfully treated with cerebrospinal fluid diversion (CFD), Ketoconazole and low molecular weight heparin. This study describes the different pathophysiological and therapeutic features of this exceptional pathogenic association.

## Introduction

Les causes générales et locales des thromboses veineuses cérébrales (TVC) ont été rapportées dans la littérature [1]. Cependant l'apparition d'une hydrocéphalie suite à une TVC est rare et n'est pas encore complètement élucidée [2]. Les localisations intracrâniennes d'*Aspergillus fumigatus* sont inhabituelles. Nous rapportons ici le cas d'un patient de sérologie VIH négative traité chirurgicalement avec succès d'une infection de la voûte crânienne à *Aspergillus fumigatus* compliquée d'une TVC, elle-même responsable d'une hypertension intracrânienne par hydrocéphalie. L'objectif de ce travail est de rapporter un cas d'infection crânio-cérébrale, avec des aspects étiopathologiques exceptionnels.

## Patient et observation

**Figure 1:**
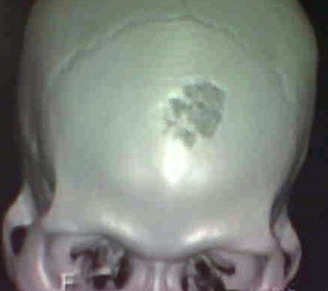
Scanner crânio-encéphalique en reconstruction 3D montrant une ostéite de la voûte frontale

**Figure 2:**
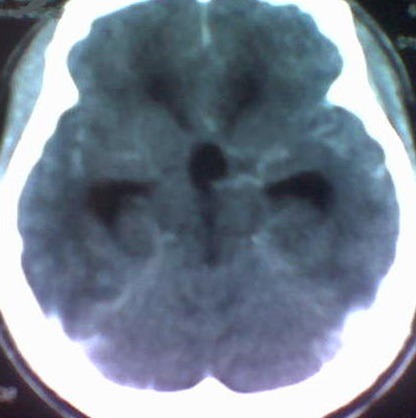
scanner cérébral avec injection en coupe axiale montrant une hydrocéphalie triventriculaire

**Figure 3:**
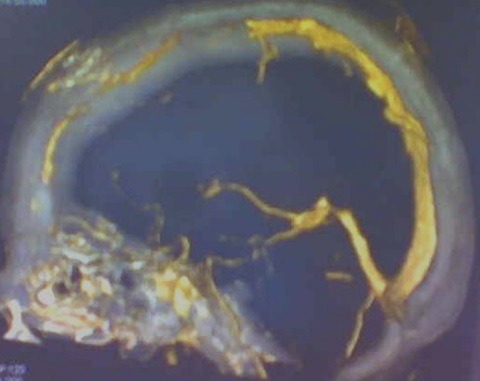
Angioscanner cérébral montrant une thrombophlébite des 2/3 antérieur du sinus sagittal supérieur

Un patient de 43 ans, sérologie VIH négative sans autres antécédents notables, a été hospitalisé dans le service de neurochirurgie du CHU de Yopougon, pour des céphalées d’intensité croissante sur une dizaine de jours. L’évolution en hospitalisation a été marquée par la survenue de vomissements spontanés puis des crises convulsives et un état confusionnel avec un délire et une agitation psychomotrice. Le tout évoluant dans un contexte d’apyrexie. L’interrogatoire a retrouvé une notion d’infection multi-récidivante du scalp frontal et d’une constipation chronique, depuis près de 5 ans. A l’examen clinique, il n’y avait pas de déficit sensitivomoteur des différents segments de membres, ni de signe méningé. L’on notait des lésions cicatricielles dyschromiques du scalp frontal. Le scanner crânio-encéphalique a objectivé une ostéite de la voûte frontale (Figure 1) avec un empyème extradural sous-jacent, une hydrocéphalie tri-ventriculaire (Figure 2). L’angioscanner cérébral montrait une thrombophlébite du sinus sagittal supérieur étendue à plus du tiers antérieur (Figure 3).

**Figure 4:**
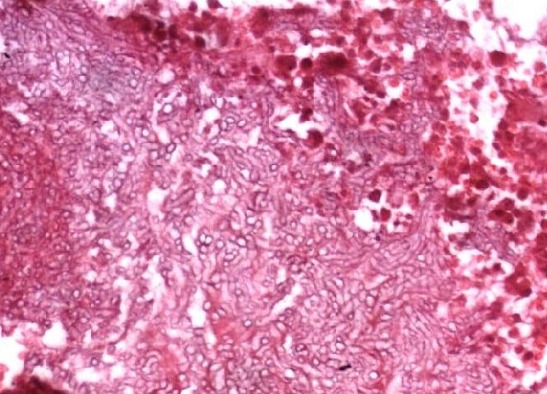
Diagnostique histologique HE x 250: feutrage dense de filaments mycéliens morphologiquement évocateur d’une aspergillose

Une dérivation du liquide cérébrospinal (LCS) par le carrefour ventriculaire, a apporté une amélioration immédiate des signes d’hypertension intracrânienne et des manifestations neuropsychiatriques. Une biopsie secondaire des lésions de la voûte frontale a permis d’isoler l’*Aspergillus fumigatus*, sensible au kétoconazole. L’examen anatomopathologique (Figure 4) a montré un feutrage dense de filaments mycéliens morphologiquement évocateur d’une aspergillose. L’analyse du LCS, montrait une hyperprotéinorachie à 1,20 g/l. A la cytologie, il y avait 2 éléments lymphocytaires. Les examens bactériologique, parasitologique et mycologique du LCS, étaient normaux, de même que la numération formule sanguine. Le traitement médical a consisté initialement, à l’association d’une tri-antibiothérapie à base d’ofloxacine, d’aminosides et de métronidazole. Secondairement, cette antibiothérapie a été remplacée par le kétoconazole après le résultat des examens microbiologiques. Il a été également associé à ce traitement, de l’héparine de bas poids moléculaire à dose curative, et un anticonvulsivant. Après 3 semaines d’amélioration neurologique, l’évolution a été marquée par la survenue d’un syndrome occlusif sur un processus tumoral du colon droit, dépisté sur les radiographies de l’abdomen sans préparation, et confirmé par une échographie abdominale.

## Discussion

La survenue d'une hydrocéphalie secondaire à une TVC, est théoriquement admise par rapport au mécanisme de résorption du liquide cérébrospinal. Cependant, c'est une circonstance exceptionnelle par rapport aux autres mécanismes d'hydrocéphalies [2]. De nombreux auteurs affirment que la TVC n'entraine généralement pas d'hydrocéphalie. Dans une publication “the *Cerebral Venous Sinus Thrombosis Study Group*” sur une étude prospective d'une série de 59 patients, aucune hydrocéphalie secondaire n'a été notée [3]. Dans notre observation l'ostéite de la voûte à *Aspergillus fumigatus* et la thrombose du sinus sagittal supérieur sous-jacente, semblent être une extension par contiguïté des lésions du scalp. De nombreuses affections, extra et intracrâniennes, peuvent être responsables de TVC. Un bilan étiologique approfondi est indispensable car la cause peut nécessiter un traitement spécifique. La cause reste indéterminée dans environ 20-30% des cas après un bilan étiologique exhaustif [1]. Le diagnostic de TVC « idiopathique » doit être posé avec extrême prudence car la cause peut être décelée uniquement lors du suivi. Les causes locales de TVC d'origine septique ont considérablement diminué depuis l'introduction des antibiotiques et dans la majorité des séries récentes cette cause n'est plus prédominante [4]. Une TVC peut compliquer une maladie générale comme le lupus, avec ou sans syndrome des antiphospholipides, la maladie de Behcet ou la maladie de Crohn. La grossesse reste une cause fréquente de TVC, en particulier dans les pays en voie de développement, du fait de l'importance des infections et de la déshydratation. La proportion des TVC durant la grossesse et le post-partum représente 5 à 20% de l'ensemble des TVC [5] dans les pays industrialisés, alors qu'elle peut atteindre 60% dans les pays en voie de développement. Avant d'incriminer un médicament dans la survenue d'une TVC, il est nécessaire de toujours rechercher d'autres causes associées. Les contraceptifs oraux sont les médicaments le plus souvent incriminés [5].

Bien que les affections pouvant conduire à une TVC soient extrêmement variées, trois principaux mécanismes physiopathologiques sont impliqués: troubles de l'hémostase (conduisant à un état prothrombotique), stase veineuse et anomalies pariétales [6]. Deux mécanismes ont été décrits en vu d'expliquer le dysfonctionnement cérébral qui accompagne une thrombose veineuse cérébrale [2]. Premièrement, la thrombose des veines corticales entraine une stase veineuse locale et engendre une hypoxie tissulaire par diminution du débit sanguin cérébral qui entraîne à son tour une ischémie et, par là même, un œdème cytotoxique. L'atteinte de la barrière hémato-encéphalique augmente le taux de filtration capillaire qui provoque un œdème vasogénique supplémentaire. Deuxièmement, la thrombose des sinus entraîne un engorgement veineux, un obstacle à la résorption du liquide cérébrospinal au niveau des granulations arachnoïdiennes, qui sont principalement situé dans le sinus sagittal supérieur et transversal, avec pour conséquence une augmentation de la pression veineuse conduisant à un œdème cérébral et une augmentation de la pression intracrânienne. La thrombose du sinus et la résultante augmentation la pression veineuse empêche l'écoulement de LCS dans le sinus. Cette cause d'hydrocéphalie n'est généralement pas claire, mais plusieurs études ont documenté une pression intracrânienne accrue après une thrombose veineuse cérébrale sans augmentation de la taille ventriculaire [7]. Les explications hypothétiques comprennent l'absorption du LCS par des veines et lymphatiques collatéraux et l'absence d'un gradient de pression entre l'espace sous-arachnoïdien et le système ventriculaire [2, 8]. Les symptômes et signes cliniques TVC sont très variés, et il est nécessaire d'évoquer facilement une TVC pour faire un diagnostic précoce. Les TVC peuvent survenir à tout âge, avec un âge moyen de 40 ans, un sex-ratio de trois hommes pour une femme. Le mode d'installation, extrêmement variable, peut être aigu (moins de 48 heures) dans 30% des cas, subaigu (entre 48 heures et 30 jours) dans 40% des cas et chronique (plus de 30 jours) dans 30% des cas [9]. Les symptômes et signes classiques, une TVC doit être suspectée lorsqu'un patient développe des symptômes et signes associant à des degrés divers une hypertension intracrânienne (céphalées, vomissements, œdème papillaire, troubles de la conscience) et/ou un déficit neurologique focal et/ou des crises épileptiques. Les céphalées constituent le symptôme le plus fréquent, présent dans 75% des cas, et souvent révélateur de la TVC. Elles n'ont pas de caractéristique ou de profil évolutif spécifique. Un œdème papillaire est présent dans environ 50% des cas. Dans près de 40% des cas, la TVC va se manifester par l'installation progressive sur plusieurs jours ou semaines d'un tableau d'hypertension intracrânienne isolée, associant des céphalées, un œdème papillaire bilatéral et plus rarement une paralysie du VI, des acouphènes et une amaurose.

Les progrès de l'imagerie non invasive permettent actuellement un diagnostic précoce de TVC. Dans certains cas, il est nécessaire de pratiquer plusieurs examens, y compris l'angiographie cérébrale, pour affirmer le diagnostic. Le scanner cérébral sans et avec injection de contraste est encore souvent réalisé en première intention. Il reste normal chez 4 à 25% des patients ayant une TVC prouvée, en particulier en cas d'hypertension intracrânienne isolée [6, 10]. Il peut montrer des signes directs et indirects de thrombose veineuse, le plus souvent non spécifiques. Le meilleur signe direct, visible sur un scanner après injection de produit de contraste, est le signe du delta, clairement visible la deuxième semaine après le début des signes cliniques. Il est présent dans environ 25% des cas, mais la fréquence varie selon les études [9, 10]. Le signe du delta apparaît comme une aire hypodense entourée d'une prise de contraste (correspondant respectivement au thrombus et aux veines collatérales dilatées) et doit être visible sur plusieurs coupes pour être pathognomonique. L'IRM cérébrale est actuellement la méthode de référence pour le diagnostic de TVC et doit être demandée systématiquement. Les séquences habituelles sont les séquences écho de spin pondérées en T1 et T2, la séquence FLAIR pour l'étude du parenchyme, la séquence T2^*^ sensible à la présence du sang et plus récemment les séquences pondérées en diffusion et perfusion (actuellement à l'étude). L'angiographie par résonance magnétique (ARM) est une technique complémentaire de l'IRM cérébrale. Elle permet la visualisation de la circulation veineuse et d'une thrombose grâce aux séquences temps de vol (TOF) 2D ou 3D, et contraste de phase. L'angiographie cérébrale est réalisée uniquement si un doute persiste sur l'IRM. Le temps veineux doit être visualisé sur au moins deux incidences. Si un défect est observé sur une injection sélective, la carotide controlatérale est comprimée pour éliminer les défauts d'opacification. L'intérêt du dosage des D-dimères a été évalué dans la démarche diagnostique concernant les TVC [11]. Ceux-ci étaient le plus souvent élevés (>500ng/mL) lorsque le diagnostic de thrombose veineuse cérébrale était confirmé hormis chez les patients ayant des symptômes évoluant depuis plus de 3 semaines. Des Dimères normaux n'excluent pas le diagnostic de thrombose veineuse cérébrale. Ils ne permettent pas d'éliminer de façon fiable ce diagnostic en particulier devant les symptomatologies atypiques. La ponction lombaire, souvent anormale, montre volontiers une augmentation de la pression, des globules rouges ou des globules blancs et une hyperprotéinorachie. L'EEG, anormal dans 75% des cas [9], montre des signes non spécifiques (ralentissements généralisés ou parfois une activité épileptique). Le rôle des explorations ultrasonores (doppler) transcrâniennes dans les TVC reste cependant limité et sont actuellement en cours d'évaluation. L'aspergillose à *Aspergillus fumigatus* est rarement diagnostiquée chez l'homme. Elle touche essentiellement les poumons. La dissémination à distance, survient chez l'immunodéprimé. Dans notre cas, la porte d'entrée la plus probable, est l'infection multirécidivante, du scalp en regard des lésions d'ostéite de la voûte. Il se pose cependant le problème de la localisation primitive ou d'une greffe secondaire d'*Aspergillus fumigatus* sur des lésions préexistantes du scalp. L'*Aspergillus fumigatus*étant habituellement un champignon saprophyte, l'hypothèse d'une greffe aspergillaire sur des lésions préexistantes du scalp semble la plus probable. L'ostéite de la voûte et la thrombose du sinus longitudinal supérieur sous-jacente, semblent être une extension par contiguïté des lésions du scalp. La pathogénie des lésions aspergillaires, dans notre observation, semblent s'expliquer par les hypothèses suivantes: d'une part, la thermotolérance d'*Aspergillus fumigatus*, permettant son développement chez son hôte à 37° et jusqu'à 55°, d'autre part sa capacité d'adhérence à la membrane basale (via le fibrinogène, la laminine, la fibronectine), sa capacité d'induire des microlésions et des ulcérations vasculaires par le biais des toxines nécrosantes, et son tropisme vasculaire.

Le traitement des TVC a plusieurs modalités, *le traitement du processus thrombotique, le traitement étiologique et le traitement symptomatique*. Le traitement du processus thrombotique (*traitement anticoagulant*) a pour objectifs de prévenir l'extension de la thrombose à d'autres sinus, afin de permettre le développement d'une circulation collatérale et la prévention des infarctus veineux. Le risque théorique est celui d'une hémorragie massive au sein d'un infarctus, volontiers spontanément hémorragique. Deux essais thérapeutiques randomisés ont évalué le rapport bénéfice/risque du traitement anticoagulant contre placebo chez des patients ayant une TVC prouvée. Le premier essai, monocentrique [12], utilisant de l'héparine non fractionnée, intraveineuse, adaptée au poids, a été arrêté après l'inclusion de 20 patients, du fait d'un bénéfice statistique significatif en faveur de l'héparine. À trois mois, dans le groupe traité, huit patients sur dix avaient totalement récupéré et deux patients gardaient un déficit modéré. En revanche, dans le groupe contrôle, trois patients étaient décédés, six avaient un déficit modéré et un seul avait récupéré totalement. Avant la mise en route du traitement, trois patients dans le groupe traité et deux dans le groupe contrôle avaient un infarctus hémorragique sur le scanner. Aucune hémorragie symptomatique n'a été observée dans le groupe traité par héparine, alors que trois patients ont eu une hémorragie dans le groupe contrôle (deux de ces patients n'avaient pas d'hémorragie initiale). Si ces résultats sont encourageants, l'étude a fait l'objet de quelques critiques méthodologiques, comme un début tardif du traitement après le début des symptômes (en moyenne un mois). Un autre essai plus récent, européen et multicentrique [13], portant sur 59 patients (30 traités, 29 placebo), a été mené à terme. Les patients traités recevaient une héparine de bas poids moléculaire (nadroparine) en sous cutanée (180 anti-Xa/kg/24 heures) pendant trois semaines suivie de trois mois d'anticoagulants oraux. À trois semaines, six des 30 patients (20%) traités avaient une évolution défavorable (définie par un décès ou un index de Barthel inférieur à 15) contre sept sur 29 dans le groupe contrôle (24%). À trois mois, une évolution défavorable était constatée respectivement dans 13 et 21%. Une récupération complète était présente respectivement dans 12% et 28%. Aucune de ces différences n'était statistiquement significative. La même proportion de patients avait un infarctus hémorragique avant traitement (respectivement 50 et 48%). Aucun patient n'a eu d'hémorragie symptomatique. Ce deuxième essai est donc globalement négatif et il paraît difficile d'attribuer cette différence aux modalités d'administration des anticoagulants. Il est toutefois intéressant de noter que 13 % des patients traités avaient une hypertension intracrânienne, contre 28% dans le groupe contrôle, ces patients ayant classiquement un bon pronostic. Une méta-analyse de ces études [13] a montré un bénéfice du traitement anticoagulant sur le pronostic vital et fonctionnel du patient, même s'il est modeste (et non statistiquement significatif).

Le risque d'hémorragie sévère au sein d'un infarctus veineux hémorragique est faible. En pratique, il est justifié de proposer un traitement anticoagulant chez tous les patients ayant une TVC certaine, y compris en cas d'infarctus hémorragique, dès lors qu'il n'existe pas de contre-indication à ce traitement. La durée optimale du traitement anticoagulant n'est pas précisément connue (trois à six mois) [14]. Les fibrinolytiques ont été testés en dehors d'essais thérapeutiques contrôlés par injection directe dans le sinus thrombosé [15], ou plus rarement par voie intraveineuse. La récupération clinique et la restauration du flux ont été rapportées avec un risque de transformation hémorragique qui semble modéré. En l'absence d'essai thérapeutique et de preuve d'une efficacité supérieure à celle de l'héparine, les fibrinolytiques ne sont envisagés que chez les patients qui s'aggravent malgré un traitement anticoagulant et symptomatique bien conduit et qui ont une extension de leur thrombose veineuse. Le traitement étiologique consiste à traiter la maladie à l'origine de la thrombose veineuse. Il doit être mis en place au plus vite, en particulier dans les cas des thromboses veineuses cérébrales septiques comme dans notre observation par l'administration antifungique. Le traitement symptomatique s'agit essentiellement du traitement de l'hypertension intracrânienne, comportant un traitement médicamenteux (Comme les corticoïdes, le mannitol, l'acétazolamide) et/ou la réalisation de ponctions lombaires soustractives. Le traitement antiépileptique, systématique en cas de crises épileptiques, peut se discuter à visée prophylactique en cas d'œdème majeur. L'évolution clinique et le pronostic des TVC sont imprévisibles à titre individuel. En effet, certains patients peuvent être initialement dans le coma et survivre sans séquelles, alors que d'autres peuvent présenter des symptômes mineurs puis s'aggraver et garder de lourdes séquelles. Le taux de mortalité dans les pays développés est d'environ 10 à 20% [9, 16], alors qu'il était de 30 à 50% dans les années 1960. Chez les survivants, le pronostic fonctionnel est meilleur que dans l'ischémie artérielle, avec 10 à 20 % des patients gardant des séquelles [9] (épilepsie, déficit focal, atrophie optique). Grâce à l'amélioration des possibilités diagnostiques et du traitement précoce, le pronostic de la TVC s'est nettement amélioré ces dernières années. Les patients atteints de TVC ont le plus souvent une récupération sans séquelle, cela a été confirmé par l'étude multicentrique récente ISCVT (International Study on Cerebral Vein and Dural Sinus Thrombosis) avec un taux de mortalité à la phase aigüe est de 4,3% [17]. Les facteurs prédictifs de décès mis en évidence par analyse multi-variée sont [18]: le coma à l'admission (score de Glasgow < 9); la confusion; les crises d'épilepsie.

## Conclusion

La thrombose veineuse cérébrale est une affection vasculaire thrombotique peu fréquente dont la clinique est polymorphe tant dans son mode de début que dans son expression symptomatique à la phase d'état. Son diagnostic implique le recours aux examens neuroradiologiques: la TDM, angioscanner, IRM et angio-IRM veineuse. Son diagnostic impose la mise en œuvre en urgence d'un traitement anticoagulant à dose curative ainsi que la réalisation d'un bilan étiologique exhaustif à la recherche d'une thrombopathie constitutionnelle ou acquise mais également d'une cause locorégionale infectieuse ou non. L'*Aspergillus fumigatus* est une étiologie inhabituelle, mais curable d'ostéite de la voûte crânienne. Elle nécessite une exploration microbiologique particulière qu'il faut savoir prescrire en présence de toute lésion inflammatoire crânio-cérébrale chronique non spécifique.

## Conflits d’intérêts

Les auteurs ne déclarent aucun conflit d'intérêts.
